# Succinyl-CoA Synthetase: New Antigen Candidate of *Bartonella bacilliformis*

**DOI:** 10.1371/journal.pntd.0004989

**Published:** 2016-09-14

**Authors:** Cláudia Gomes, Noemí Palma, Maria J. Pons, Ariel Magallón-Tejada, Isabel Sandoval, Carmen Tinco-Valdez, Carlos Gutarra, Juana del Valle-Mendoza, Joaquim Ruiz, Mayumi Matsuoka

**Affiliations:** 1 ISGlobal, Barcelona Centre for International Health Research (CRESIB), Hospital Clínic, Universitat de Barcelona, Barcelona, Spain; 2 Centro de Investigación e Innovación, Facultad de Ciencias de la Salud, Universidad Peruana de Ciencias Aplicadas, Lima, Peru; 3 Red de Salud de Morropon Chulucanas, Piura, Peru; 4 Instituto de Investigación Nutricional, Lima, Peru; 5 National Institute of Infectious Diseases, Tokyo, Japan; Komfo Anokye Teaching Hospital, GHANA

## Abstract

**Background:**

*Bartonella bacilliformis* is the causative agent of Carrion’s disease, a neglected illness with mortality rates of 40–85% in the absence of treatment. The lack of a diagnostic technique to overcome misdiagnosis and treat asymptomatic carriers is of note. This study aimed to identify new *B*. *bacilliformis* antigenic candidates that could lead to a new diagnostic tool able to be implemented in endemic rural areas.

**Methodology/Principal Findings:**

Blood (n = 198) and serum (n = 177) samples were collected in northern Peru. Clinical data were recorded. Specific *16S rRNA* amplification by RT-PCR, IFA and ELISA for IgM/IgG with whole cells as antigens was done. Western blot analysis and N-terminal amino acid sequencing detected seroreactive proteins. ELISAs for IgM/IgG for the antigenic candidates were performed. Of the population 33.3% reported at least one symptom compatible with Carrion’s disease; 25.4% (IFA), 27.1% (ELISA-IgG), 33.9% (ELISA-IgM) and 38.9% (RT-PCR) of samples were positive. Four proteins were considered potential antigenic candidates, including two new antigenic candidates, succinyl-CoA synthetase subunit α (SCS-α) and succinyl-CoA synthetase subunit β (SCS-β). On Western blot both Pap31 and SCS-α interacted with IgM, while GroEL and SCS-β interacted with IgG. The presence of specific antibodies against the antigenic candidates varied from 34.5% (IgG against SCS-α) to 97.2% (IgM against Pap31).

**Conclusions/Significance:**

RT-PCR and the high levels of positivity for specific ELISAs demonstrate high levels of *B*. *bacilliformis* exposure and asymptomatic carriers among inhabitants. The new antigens identified might be used as a new rapid diagnostic tool to diagnose acute Carrion’s disease and identify asymptomatic carriers.

## Author Summary

*B*. *bacilliformis* is a neglected pathogen causing Carrion’s disease, a febrile illness with two distinct phases, the acute so-called Oroya fever that can be life-threatening, and the chronic so-called Peruvian wart. This illness is currently limited to poor inhabitants of Andean valleys of Ecuador, Colombia and Peru and for this reason is understudied. One of the most significant limitations is the lack of an adequate diagnostic tool able to be implemented in rural areas. It is imperative to unequivocally detect cases of Carrion’s disease as well as identify asymptomatic carriers who perpetuate the illness. The present study describes the identification of 4 antigenic candidates potentially useful in the future development of a rapid diagnostic test. Moreover, 2 of these candidates have not been described in the literature. Additionally, four post-outbreak and one endemic community were studied and characterized. The identification of new antigens is essential for the development of a cheap, sensitive diagnostic tool, able to be implemented in low-income areas.

## Introduction

*Bartonella bacilliformis* is the etiological agent of Carrion’s disease, a neglected endemic illness in Peru which has also been reported in Ecuador and Colombia [1]. Two well-established phases have been described in this infection. In the acute phase, also called Oroya Fever, *B*. *bacilliformis* infects the red blood cells which may result in severe anemia and transient immunosuppression [2,3]. The absence of treatment leads to high levels of mortality (40% to 85%) [4]. The chronic phase, *‘Verruga Peruana’* (Peruvian wart), is characterized by the development of nodular dermal eruptions. This phase typically occurs in survivors weeks or months after the acute febrile syndrome [5].

Clinical cure does not necessarily result in bacterial clearance. In fact, viable *B*. *bacilliformis* have been cultured from blood samples of treated patients [6,7]. This lack of clearance together with the development of partial immunity and the presence of continuous *B*. *bacilliformis* exposure, means that endemic areas have a high number of individuals who are asymptomatic carriers. Indeed, it has been described that 45% of inhabitants of endemic areas are *B*. *bacilliformis* seropositive when antibodies are tested by Indirect Fluorescence Antibody (IFA) assay [8].

Studies of *B*. *bacilliformis* antigens are scarce in the literature compared with reports of other pathogens, and a rapid diagnostic method to detect acute and/or chronic infections has yet to be developed. To our knowledge, the first report identifying *B*. *bacilliformis* antigens was described in 1988 by Knobloch [9]. Twenty-four protein antigens were found, including one main antigen with 65 kDa (BB65; a heat shock protein posteriorly identified as GroEL) [9–11]. Nonetheless, BB65 never bound to *Bartonella* IgM but did bind to IgG antibodies after the first two weeks, thereby demonstrating its utility to detect persisting IgG from the first to the third year after a *B*. *bacilliformis* infection. However, only 60% of sera from Verruga Peruana patients react with BB65 [10]. Padmalayam *et al*. described an immunogenic 43 kDa lipoprotein as an antigen by screening a genomic DNA lambda library with serum from a patient in the chronic wart phase of bartonellosis [12]. More recently, Pap31 was described to be immunologically dominant and a good candidate for use in ELISA, but once again only one serum was used for identification [13]. Unfortunately, none of the antigens described above have resulted in a rapid diagnostic tool.

The objective of this study was to identify and characterize *B*. *bacilliformis* antigenic candidates and take a step towards a rapid diagnostic tool able to be implemented in rural areas.

## Materials and Methods

### Bacterial strains

The microorganisms used in this study are listed in Table 1. *B*. *bacilliformis* was cultured at 28°C on Columbia agar with 5% sheep blood for 7 days.

**Table 1:** Bacterial strains and plasmids.

	Description	Source or reference
Strains		
*B*. *bacilliformis*	CIP 57.20 –NCTC12136	Institute Pasteur
E. coli TOP10	Host strain for cloning	Invitrogen
E. coli BL21Star (DE3)	Host strain for gene expression	Invitrogen
Plasmids		
pCR4-TOPO	TA-cloning vector	Invitrogen
pET100D/TOPO	Expression vector	Invitrogen
pGroEL	pET100D/TOPO containing GroEL	This study
pPap31	pET100D/TOPO containing Pap31	This study
pSCS-α	pET100D/TOPO containing SCS-α	This study
pSCS-β	pET100D/TOPO containing SCS-β	This study

SCS-α: succinyl-CoA synthetase subunit α

SCS-β: succinyl-CoA synthetase subunit β.

### Study population

Blood (n = 198) and serum samples (n = 177) were collected in March 2014 in five different villages of Piura, in the north of Peru: Tunal, Guayaquiles, Los Ranchos, Mayland and Huancabamba. Serum samples were collected in sodium citrate and gel SSTII advance vacutainers (BD, Heidelberg, Germany). A *B*. *bacilliformis* outbreak occurred in 4 of these villages: between May and November 2013 in Tunal, Guayaquiles and Mayland, and between November 2013 and March 2014 in Los Ranchos [14]. According to the national guidelines [15], all participants had received ciprofloxacin treatment during 14 days following diagnosis during the outbreak. All people living in the aforementioned four villages who were diagnosed (clinical symptoms and/or thin blood smear) with acute Carrion’s disease during the previous outbreak were invited to participate in the study. Those who agree to participate were notified by the local health center and all samples from that village were collected on the same day. On the other hand, Huancabamba is a long established endemic area for Carrion’s disease and the volunteers were randomly recruited by house-to-house visits. Clinical and demographic data were recorded in all cases. After collection the samples were kept in the appropriate refrigeration conditions: 4°C for blood samples and sera samples were frozen. All samples were transferred to Lima (Peru) and sera samples were also sent to Tokyo (Japan) to perform immunological assays. In all cases transportation was performed under frozen conditions.

### Ethics statement

In all the cases, signed informed consent was obtained and clinical data were anonymized. In the case of children a parent or their guardian provided informed consent on their behalf. The study was approved by both the Ethic Committees of the Universidad Peruana de Ciencias Aplicadas (Lima, Peru) and the Hospital Clinic (Barcelona, Spain).

### Real-Time (RT) PCR

DNA extraction was done from 200 μL blood samples with the Qiamp DNA Mini Kit (Qiagen, Hilden, Germany), according to the manufacturer’s instructions except that the final elution volume was 100 μL. A RT-PCR was performed based on Smit *et al*. [16], using a BHQ quencher probe at 125 nM and 250 nM of primers in final volume of 25 μL. RT qPCR conditions were 95°C for 15 minutes, 60 cycles of 15 seconds at 95°C and 45 seconds at 60°C and the procedure was performed in an ABI Prism 7500 RT system and data was analyzed in the 7500 System SDS software (Applied Biosystems, Warrington, UK). The primers and the probe used are shown in Table 2.

**Table 2:** Primers used in this study.

	Sequence (5’ → 3’)	Ref
*16S rRNA* F	TTGATAAGCGTGAGGTCGGAGG	[16]
*16S rRNA* R	GCAACCACACATAGTAAGCCTAA	[16]
*16S rRNA* probe	ATGTCTGCCTAGAAATCAATCATAGGCC	[16]
GroEL F	*CACCATGGCTGCTAAAGAAGTAAAATTT	This study
GroEL R	TTAGAAATCCATTCCGCCCATTCCGCC	This study
Pap31 F	*CACCATGAATATAAAATGTTTAGTGACA	This study
Pap31 R	TCAGAATTTGTAAGCAACACCAACGCG	This study
SCS α F	*CACCATGTCAATTCTTATC	This study
SCS α R	CTAACCCTTCAAGACTGAAACC	This study
SCS β F	*CACCATGAATATCCATGAAT	This study
SCS β R	TTAAGCTCCTTTTACGGCTGC	This study

* CACC at the 5’ end is a sequence added to allow pET directional cloning.

### IFA assay

The Chamberlin method was used with slight modifications [8]. Briefly, Vero cells were cultured in sterile plates maintaining the same MOI and concentrations throughout the experiment. At harvest, the Vero cell monolayer was removed with trypsin in a final volume of 10 mL. The slides were mounted with 65 μL of cells suspension per well and 20 μL 1:100 of Fluorescein (FITC)-conjugated goat anti-human IgG (heavy plus light chains) (Jackson IR, Baltimore, PA). Slides were read using a 20X objective, 10X oculars and an Olympus IX51 microscope. We used the same cut off established by Chamberlin *et al*., considering 1/256 serum dilution end point as a positive IFA test.

### ELISA

IgM and IgG antibody levels were measured by ELISA as described by Matsuoka *et al*. [17] using sonicated *B*. *bacilliformis* whole cells as antigens. Total protein concentration was quantified by the Pierce assay (Thermo Scientific, Rockford, USA). IgM were detected with rabbit anti-human IgM (1:1000) conjugated with peroxidase and using o-Phenylenediamine (Dako, Glostrup, Denmark) as substrate (Sigma, St. Louis, MO). IgG were detected with rabbit anti-human IgG (1:1000) conjugated with alkaline phosphatase and using Sigma P104 phosphatase substrate. Optical densities were measured as absorbance at 450 nm and 405/655 nm for IgM and IgG, respectively. Each sample was analyzed in triplicate intra-plate and results were reported as three independent ELISA experiments. The negative control, included a pool of sera from 30 healthy donors (X0939, Dako, Glostrup, Denmark). In the absence of an established cut off, evidence of infection was considered in the samples above the Finite Mixture Model (FMM) cut off that provides a statistical framework for the analysis of immunoglobulin values [18].

### Western blotting

Agar-grown *B*. *bacilliformis* were lysed by sonication, separated on a 10 to 20% SDS-PAGE gel and electrotransferred onto a PVDF membrane (GE Healthcare, Buckinghamshire, UK). The different lanes were cut and immunoblotted with each serum for both IgG/IgM. The negative control used in ELISA was used here.

### Amino acid sequencing

Immunogenic candidate proteins were chosen according to the antibody levels obtained by whole cell ELISA results. The membranes were stained with Coomasie brilliant blue staining solution. Candidate proteins were directly cut out, and N-terminal amino acid sequencing was done in APROScience (Naruto, Japan).

### Amplification and cloning of antigenic candidates

Genes coding for the candidate proteins were amplified by PCR using the primers in Table 2. Amplified products were purified and cloned in Champion pET Directional 100/D-TOPO vector (Invitrogen, Carlsbad, CA), according to the manufacturer’s instructions in order to generate Xpress-tagged-full-length versions of the candidate antigenic proteins. The DNA sequences were verified. For the detection, *E*. *coli* lysates suspended in lysis buffer were separated on a 12.5% SDS-PAGE and immunoblotted with anti-Xpress antibody. The His_6_-tagged proteins were purified using His-Bind kits (GE Healthcare, Buckinghamshire, UK).

### Candidate antigens ELISA

Purified candidate proteins were used to perform ELISA and determine the reaction with sera in study, following the same protocol mentioned previously. Here, the cut off was established considering 3 times the standard deviation for the X0939 negative control (Dako).

### Statistical analysis

Statistical analysis was performed using the Graphpad package. Significance was considered with *P*<0.05. *P*-values were calculated using the Fisher and Mann-Whitney tests.

## Results

A Carrion’s disease outbreak occurred in the Lalaquiz district (S1 Fig) between May and November 2013. Sixty-five villages were affected, with Tunal, Guayaquiles and Mayland being among the 10 most affected with 53.8, 19.3 and 2.56% of the cases, respectively. Meanwhile, in Los Ranchos (in the neighboring Canchaque district) 21 cases were reported between November 2013 and March 2014. This outbreak was the first description of this illness in these villages. During the outbreak, a total of 428 cases were reported, diagnosed by clinical symptoms and/or thin blood smear. The mean age of the persons affected during the outbreak was 34 years; 52.3% of the cases were females, and 18.5% of the cases involved children up to 10 years of age. Headache (74.7%), malaise (66%), fever (39.2%), arthralgia (31.6%), myalgia (24.4%) and pallor (13%) were the most common symptoms.

We were able to collect 198 blood samples from volunteers in March 2014 and Table 3 shows the study population data. All samples collected in the 4 post-outbreak villages were from people who had been diagnosed with acute Carrion’s disease a few months earlier when an outbreak had occurred. Despite the fact that all volunteers appeared healthy, mild symptoms were reported. The most common symptom described in our study population was headache in 27.3% of the population, followed by 4% of people reporting fever, myalgia and malaise. Joint pain and pallor were present in 5 and 2.5%. With regard to the endemic village, 1 (0.5%) person from the endemic area (Huancabamba) presented Verruga Peruana at the time of sample collection. RT-PCR results showed amplification for 77 (38.9%) individuals, but it should be taken into account that all positive samples presented very low bacteremia in blood. Significant differences were found between the positivity results of Tunal and Guayaquiles (*P* = 0.0189), Guayaquiles and Los Ranchos (*P* = 0.0370), Tunal and Mayland (*P* = 0.0006), Los Ranchos and Mayland (*P* = 0.0001) and Los Ranchos and Huancabamba (*P* = 0.0178). Additionally, the results between Mayland and Huancabamba were almost statistically significant (*P* = 0.0548) (Table 4).

**Table 3:** General data about the study population (n = 198).

	Tunal	Guayaquiles	Los Ranchos	Mayland	Huancabamba	Total
	(n = 94)	(n = 25)	(n = 44)	(n = 10)	(n = 25)	
n (%)	94 (47.5)	25 (12.6)	44 (22.2)	10 (5.1)	25 (12.6)	198 (100)
Mean age (min–max)	39.3 (1–94)	34.2 (6–67)	38.6 (5–77)	30.2 (6–73)	30.2 (9–68)	36.9 (1–94)
Male	31 (33)	8 (32)	17 (38.6)	4 (40)	16 (64)	76 (38.4)
Female	63 (67)	17 (68)	27 (61.4)	6 (60)	9 (36)	122 (61.6)
CD symptoms*	23 (24.5)	17 (68)	14 (31.8)	3 (30)	9 (36)	66 (33.3)

*The presence of at least one symptom compatible with Carrion’s disease, including fever, joint pain, headache, malaise, pallor, myalgia and warts.

**Table 4:** Positive results for each technique in the different study sites.

		Tunal	Guayaquiles	Los Ranchos	Mayland	Post-outbreak areas	Endemic area (Huancabamba)	*P*^*1*^	Total
		n/N (%)	n/N (%)	n/N (%)	n/N (%)	n/N (%)	n/N (%)		n/N (%)
**RT-PCR**		30/94 (31.9)^a^^,^^c^	15/25 (60)^a^^,^^b^	10/44 (22.7)^b^^,^^d^^,^^e^	9/10 (90)^c^^,^^d^	64/173 (37)	13/25 (52)^e^	0.1883	77/198 (38.9)
**IFA**		21/75 (28)	8/25 (32)	8/43 (18.6)	4/10 (40)	41/153 (26.8)	3/24 (12.5)	0.0575^‡^	45/177 (25.4)
**ELISA**	**IgM**	19/75 (25.3)	9/25 (36)	17/43 (39.5)	5/10 (50)	50/153 (32.7)	10/24 (41.7)	0.4869	60/177 (33.9)
	**IgG**	14/75 (18.7)^f^	6/25 (24)^g^	10/43 (23.3)^h^	2/10 (20)^i^	32/153 (20.9)	16/24 (66.7)^f^^,^^g^^,^^h^^,^^i^	0.0001**	48/177 (27.1)
**Positive for at least one technique**^†^		55/75 (73.3)	23/25(92)	31/43 (72.1)	10/10 (100)	119/153 (77.8)	22/24 (91.7)	0.1715	141/177 (79.7)

^†^Only individuals with both blood and serum samples were considered (n = 177).

^1^Statistical significance between the positivity in the 4 post-outbreak villages taken together and the positivity in the endemic area (Huancabamba).

^‡^IFA results were almost significantly lower in the endemic area than in the 4 post-outbreak villages taken together (*P* = 0.0575).

**ELISA IgG values were significantly higher in Huancabamba than in the 4 post-outbreak villages taken together (*P* = 0.0001).

The superscript letters represent the statistically significant differences between the positivity in 2 specific areas

^a^ *P* = 0.0189

^b^ *P* = 0.0370

^c^ *P* = 0.0006

^d^ *P* = 0.0001

^e^ *P* = 0.0178

^f^ *P*<0.0001

^g^ *P* = 0.0041

^h^ *P* = 0.0007

^i^ *P* = 0.0229.

The RT-PCR values between Mayland and Huancabamba were almost statistically significant (*P* = 0.0548). In all cases the Fisher exact test was used.

**Fig 1:**
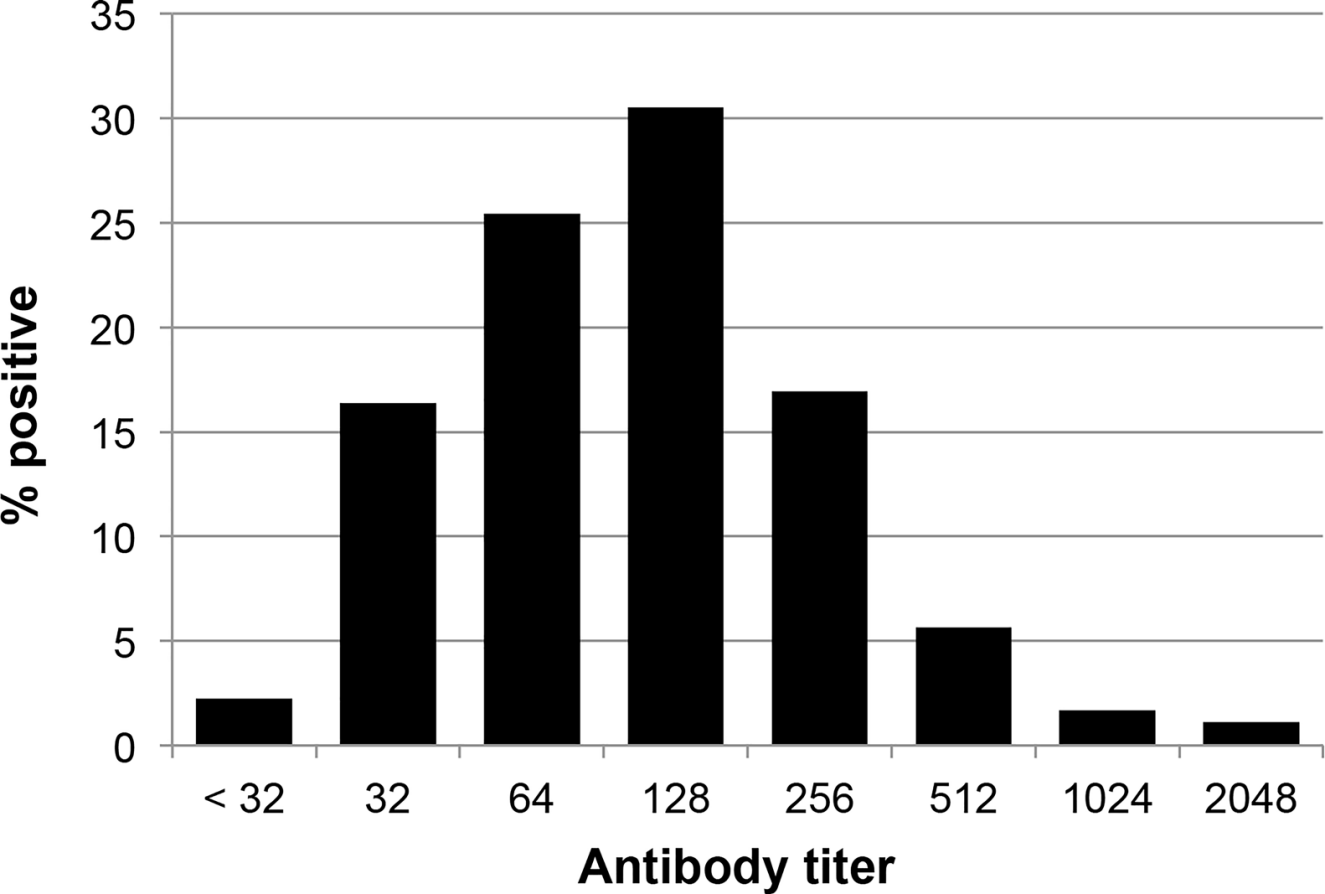
Distribution of *B*. *bacilliformis* antibodies by IFA assay among the study population (n = 177).

**Fig 2:**
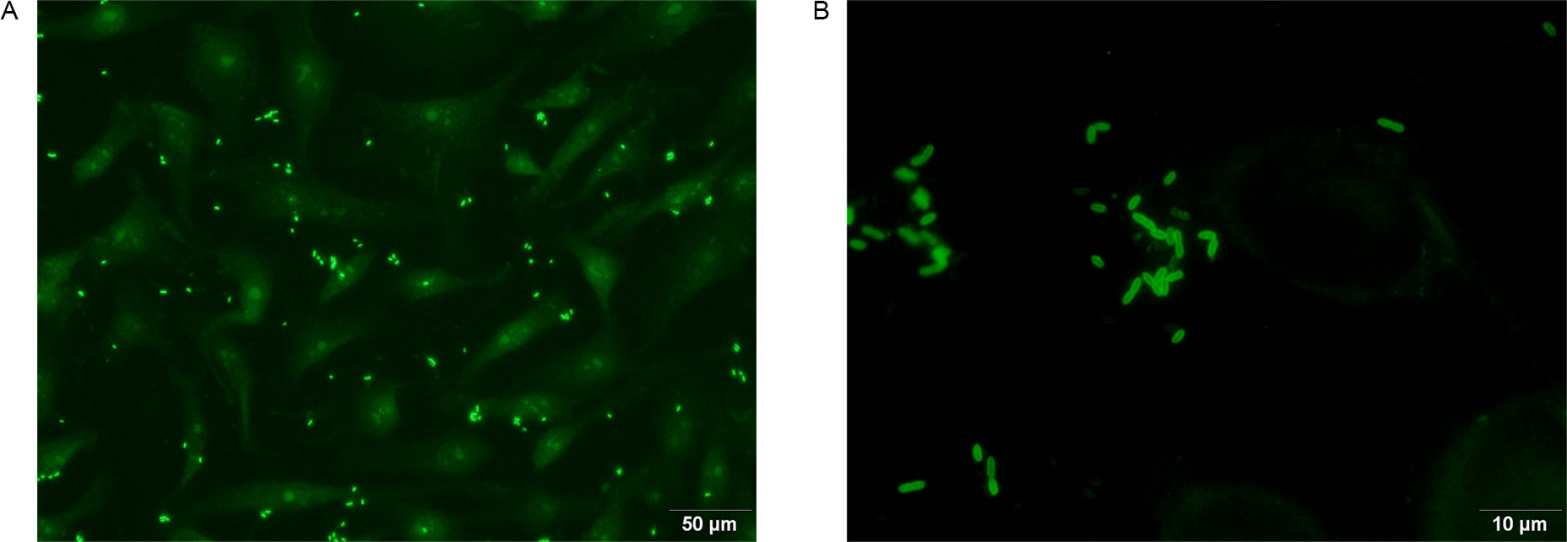
Example of a positive IFA test. A) 20X objective and 10X oculars. B) 100X objective and 10X oculars.

Serum samples were taken from 177 (89.4%) of the individuals. Of these 45 (25.4%) had an IFA titer ≥ 256 being considered as positive by the IFA test (Figs 1 and 2, Table 4). The IFA results were almost significantly lower in the endemic area than in the 4 post-outbreak villages taken together (*P* = 0.0575).

For the whole cell *B*. *bacilliformis* IgM ELISA, the 3 standard deviation cut off method resulted in 95.5% of volunteers presenting levels of IgM equivalent to evidence of infection, while with the use of FMM, the cut off was established at 0.35 (IgM) and 0.53 (IgG), with approximately 34 and 27% of volunteers showing evidence of infection by IgM and IgG values, respectively. Thus, in order to differentiate the most positive samples we applied the FMM cut off.

**Fig 3:**
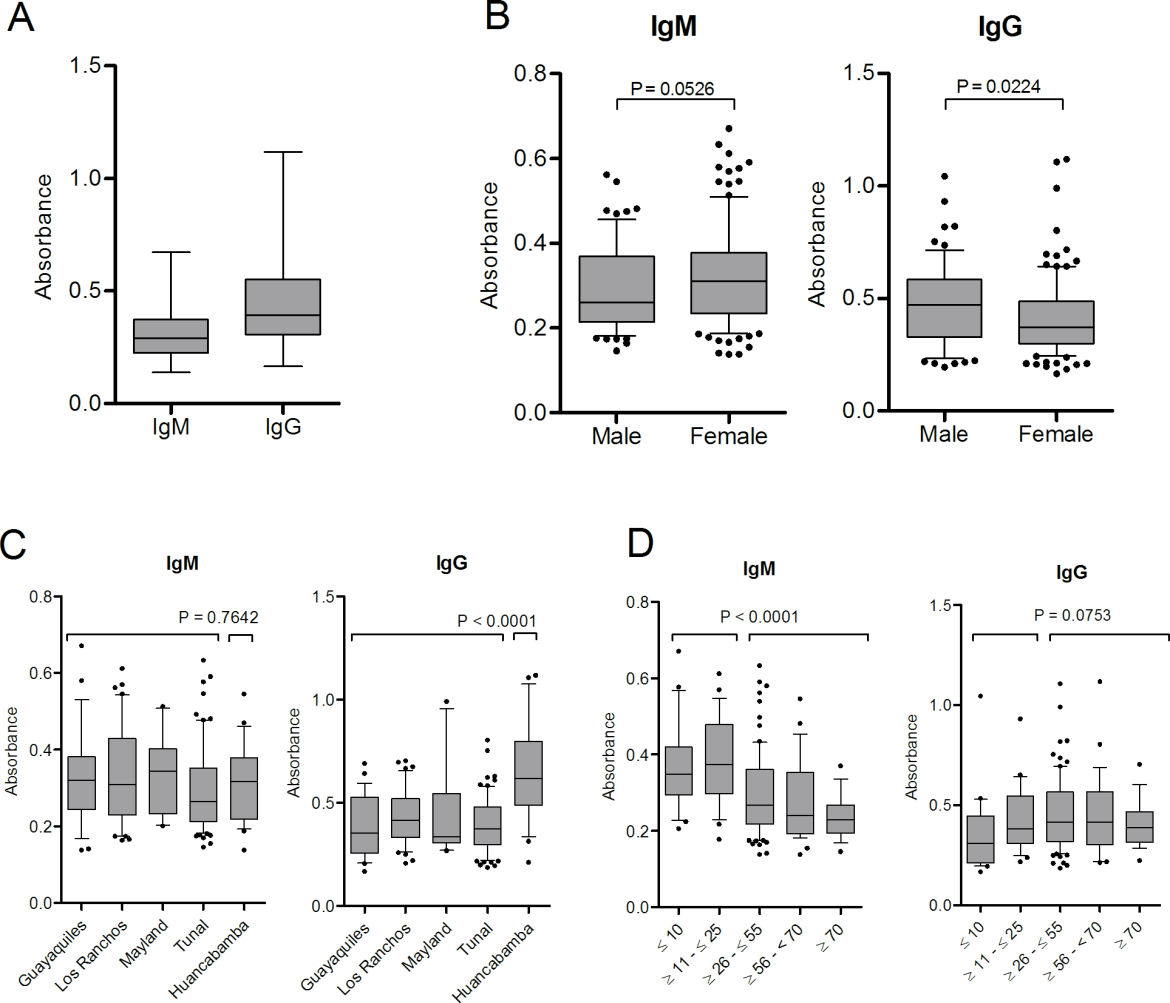
IgM and IgG antibodies in the study population. A) Overall data. Data are presented as boxplots that illustrate the medians and the maximum and minimum. B) IgM and IgG distribution by sex; C) IgM and IgG distribution by locality. *P*-values obtained on comparing the 4 post-outbreak areas with the endemic area. When comparing pairs of localities we obtained significance for IgG with the following analysis: Guayaquiles and Huancabamba (*P* = 0.0001); Los Ranchos and Huancabamba (*P* = 0.0002); Mayland and Huancabamba (*P* = 0.0201); Tunal and Huancabamba (*P*<0.0001). D) IgM and IgG distribution by age groups. *P*-values obtained comparing the antibody levels of subjects between ≤ 25 years with ≥ 26 years. In panels B, C and D the data are presented as boxplots that illustrate the medians and the 25th and 75th quartiles, and the whiskers represent the 10% and 90% percentiles. Outliers are marked with circles. *P*-values were calculated using the Mann-Whitney test.

Differences in the antibody levels between genders were not found. However, it was observed that higher IgM levels tended to be more frequent in females (*P* = 0.0526) while IgG values were significantly higher in men (*P* = 0.0224). On analyzing the antibody levels by localities, we saw that IgG levels were significantly higher (*P*<0.0001) in Huancabamba compared to post-outbreak areas where the disease had never been described before this outbreak. This shows the presence of a high percentage of healthy people with high levels of antibodies against *B*. *bacilliformis*. Additionally, for IgG levels statistically significant differences (*P* values ranging from <0.0001 to 0.0229) were obtained on comparing each of the post-outbreak areas with the endemic area (Huancabamba) (Table 4). When the IgM levels were analyzed taking into consideration the different age groups, we observed that young people (≤25 years) had significantly higher IgM levels (*P*<0.0001) than adults ≥26 years, while IgG levels only showed a tendency to be higher in people ≥26 years (*P* = 0.0753). On comparing the IgG levels of the younger population (≤10 years) with each of the other age groups, we found statistically significant differences with all groups except people ≥70 years (between ≥11 - ≤25 *P* = 0.0281; ≥26 - ≤55 *P* = 0.0074; ≥56 - ≤70 *P* = 0.0246) (Fig 3).

No concordance was observed between clinical data and the positivity obtained with the diagnostic tools used. It is interesting to note that the case of Peruvian wart was only detected by ELISA IgG (Table 5).

**Table 5:** Distribution of the positive individuals for each diagnostic technique used according to the symptoms described.

	N (%)*	RT	ELISA_IgM	ELISA_IgG	IFA
		n (%)*	n (%)*	n (%)*	n (%)*
**Headache**	48 (27.1)**	23 (47.9)	10 (20.8)	13 (27.1)	15 (31.3)
**Joint pain**	10 (5.6)	3 (30.0)	2 (20.0)	2 (20.0)	4 (40.0)
**Fever**	8 (4.5)	4 (50.0)	2 (25.0)	3 (37.5)	4 (50.0)
**Malaise**	8 (4.5)	4 (50.0)	2 (25.0)	3 (37.5)	4 (50.0)
**Myalgia**	8 (4.5)	4 (50.0)	2 (25.0)	4 (50.0)	3 (37.5)
**Pallor**	5 (2.8)	4 (80.0)	2 (40.0)	1 (20.0)	4 (80.0)
**Peruvian wart**	1 (0.6)	0	0	1 (100.0)	0
**Any symptom**	60 (33.9)	27 (45.0)	13 (21.7)	17 (28.3)	18 (30.0)

* percentage in the population (considering n = 177).

** 6 more individuals presented headache but were not considered in this table because only the blood sample were collected.

Table 6 shows the concordance between each pair of techniques used. Six samples had a positive result for at least 3 out of the 4 techniques used. This comparative analysis showed a certain degree of dispersion and a lack of concordance (Table 6).

**Table 6:** Concordance among the techniques used.

		RT-PCR		IFA		ELISA IgM	
		N (%)		N (%)		N (%)	
		+	-	+	-	+	-
IFA	+	20 (11.3)	24 (13.6)				
	-	53 (29.9)	80 (45.2)				
ELISA IgM	+	24 (13.6)	36 (20.3)	14 (7.90)	46 (26.0)		
	-	49 (27.7)	68 (38.4)	30 (16.9)	87 (49.2)		
ELISA IgG	+	19 (10.7)	29 (16.4)	9 (5.1)	39 (22.0)	19 (10.7)	29 (16.4)
	-	54 (30.5)	75 (42.4)	35 (19.8)	94 (53.1)	41 (23.2)	88 (49.7)

Only individuals with both blood and serum samples were considered (n = 177).

**Fig 4:**
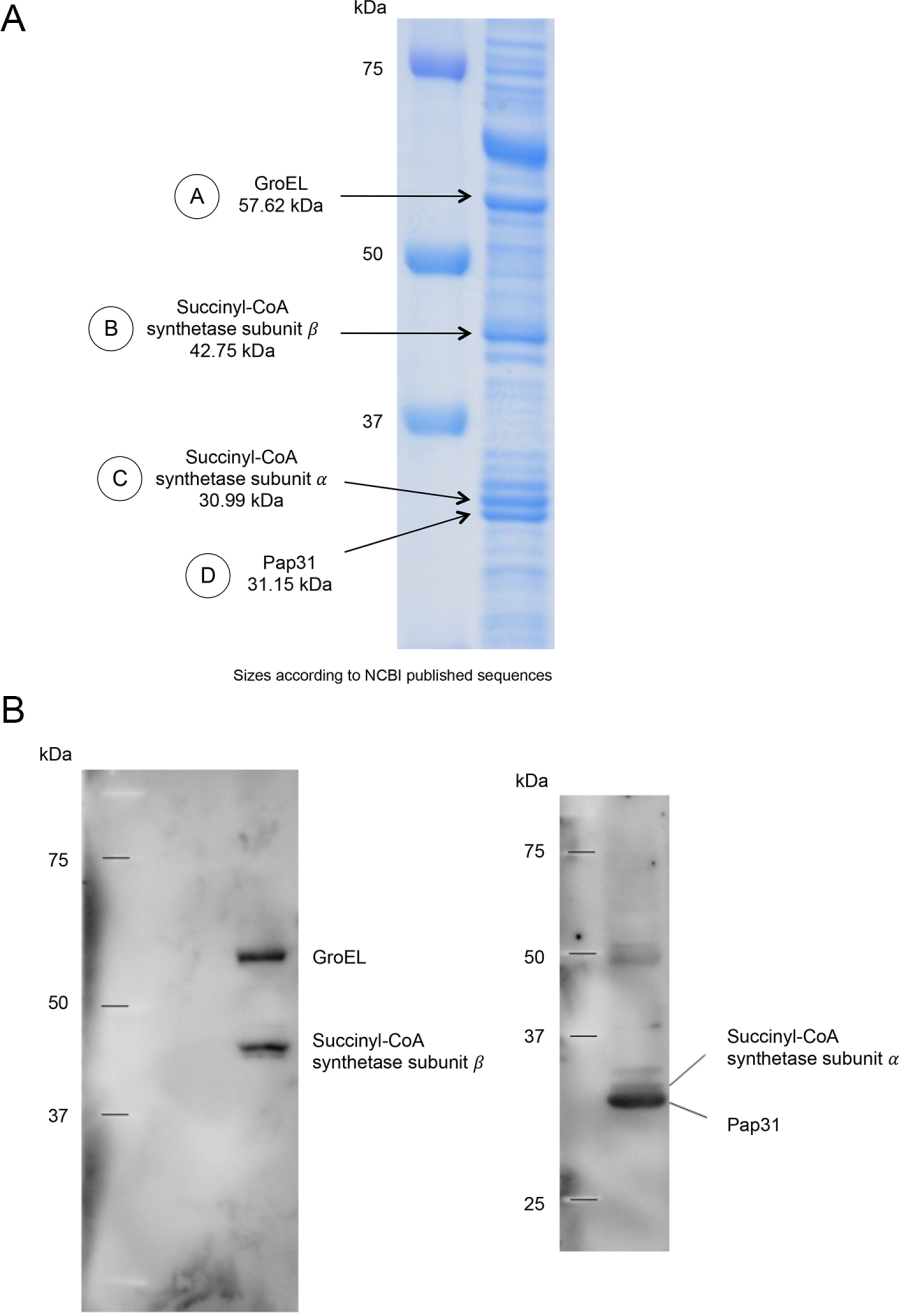
Antigenic candidates of *B*. *bacilliformis* selected. A) SDS gel with sonicated whole cell *B*. *bacilliformis*. B) Example of a Western blot with a positive serum by ELISA. The left panel corresponds to IgG and the right panel to IgM.

By doing the Western blots with the sera in the study and against whole sonicated *B*. *bacilliformis* we chose four proteins as possible antigenic candidates, two identified with anti-human IgM and two with anti-human IgG (Fig 4). Amino acid sequencing revealed that the IgM candidates identified were Pap31 and succinyl-CoA synthetase subunit α (SCS-α) whereas for IgG we identified GroEL and succinyl-CoA synthetase subunit β (SCS-β) (Table 7).

**Table 7:** N-terminal sequencing results.

Protein*	N-terminal sequence	Molecular Mass (KDa)	Homologous Protein	Identity** (%)	Accession number	Organism
A	AAKEVKFGRDARERL	57.52	GroEL	100.0	CH60_BARBK	***Bartonella bacilliformis***
		57.62	GroEL	100.0	CH60_BARBA	***Bartonella bacilliformis***
		57.62	GroEL	93.3	CH60_BARHE	*Bartonella henselae*
		57.61	GroEL	93.3	CH60_BARQU	*Bartonella quintana*
		57.60	GroEL	93.3	CH60_BART1	*Bartonella tribocorum*
						
B	MNIHEYQAKRLLHEY	42.74	SCS-β	100.0	SUCC_BARBK	***Bartonella bacilliformis***
		42.82	SCS-β	100.0	SUCC_BARHE	*Bartonella henselae*
		43.00	SCS-β	100.0	SUCC_BARQU	*Bartonella quintana*
		42.87	SCS-β	100.0	SUCC_BART1	*Bartonella tribocorum*
		42.53	SCS-β	93.3	SUCC_BRUAB	*Brucella abortus*
		42.53	SCS-β	93.3	SUCC_BRUA1	*Brucella abortus*
		42.53	SCS-β	93.3	SUCC_BRUC2	*Brucella canis*
		42.53	SCS-β	93.3	SUCC_BRUMB	*Brucella melitensis*
		42.53	SCS-β	93.3	SUCC_BRUME	*Brucella melitensis*
		42.53	SCS-β	93.3	SUCC_BRUO2	*Brucella ovis*
		42.53	SCS-β	93.3	SUCC_BRUSU	*Brucella suis*
		42.53	SCS-β	93.3	SUCC_BRUSI	*Brucella suis*
		42.52	SCS-β	93.3	SUCC_OCHA4	*Ochrobactrum anthropi*
						
C	SILINKDTKVLVQGL	30.93	SCS-α	100.0	WP_024847155	*Aminobacter* spp.
		30.99	SCS-α	100.0	BG36_06620	*Aquamicrobium defluvii*
		32.92	SCS-α	100.0	B9JCF2_AGRRK	*Agrobacterium radiobacter*
		30.90	SCS-α	100.0	B9JTS6_AGRVS	*Agrobacterium vitis*
		31.07	SCS-α	100.0	E6YT34_9RHIZ	*Bartonella* spp.
		30.99	SCS-α	100.0	E6YXC2_9RHIZ	*Bartonella* spp.
		30.99	SCS-α	100.0	WP_035454724	***Bartonella bacilliformis***
		30.99	SCS-α	100.0	A1UQW0_BARBK	***Bartonella bacilliformis***
		31.01	SCS-α	100.0	E6YNR1_9RHIZ	*Bartonella rochalimae*
		30.96	SCS-α	100.0	WP_026620040	*Ensifer* spp.
		30.90	SCS-α	100.0	A9DG17_HOEPD	*Hoeflea phototrophica*
		31.08	SCS-α	100.0	WP_027835951	*Maritalea myrionectae*
		30.97	SCS-α	100.0	WP_028751285	*Rhizobium leucanae*
		30.99	SCS-α	100.0	L0LRG5_RHITR	*Rhizobium tropici*
		30.90	SCS-α	100.0	WP_026615514	*Sinorhizobium* spp.
		30.93	SCS-α	100.0	WP_028001748	*Sinorhizobium arboris*
		30.87	SCS-α	100.0	G9A287_RHIFH	*Sinorhizobium fredii*
		30.97	SCS-α	100.0	I3XDY7_RHIFR	*Sinorhizobium fredii*
		30.91	SCS-α	100.0	A6UDP1_SINMW	*Sinorhizobium medicae*
		30.93	SCS-α	100.0	F7X0D4_SINMM	*Sinorhizobium meliloti*
						
D	ADVMIPQEISPIISA	31.70	Pap31	100.0	Q3HM50_BARBA	***Bartonella bacilliformis***
		31.16	Pap31	100.0	WP_035454931	***Bartonella bacilliformis***
		31.61	Omp25	100.0	A1UU13_BARBK	***Bartonella bacilliformis***

* As indicated in Fig 4.

** Only those with identity levels > 90%.

After cloning, expression and purification of the four antigenic candidates, we performed ELISA for each IgM and IgG testing.

The IgM results showed a high prevalence of reactivity, from 77.4% for SCS-α to 97.2% for Pap31. Meanwhile, the IgG reactivity in the ELISAs done for the antigens ranged from 34.5 to SCS-α and 59.9% to SCS-β IgG (Table 8). The person with the Verruga Peruana was positive for IgG anti-GroEL, but no other specific association was observed between symptoms and other IgM/IgG positive status.

**Table 8:** Positive prevalence for the antigenic candidate ELISAs.

	SCS-α*	Pap31	SCS-β*	GroEL
	N (%)	N (%)	N (%)	N (%)
IgM	137 (77.4)	172 (97.2)	168 (94.9)	138 (78.0)
IgG	61 (34.5)	104 (58.8)	106 (59.9)	70 (39.5)

*Succinyl-CoA synthetase. Cut offs considered: SCS-α (IgM) = 0.076; SCS-α (IgG) = 0.130; Pap31 (IgM) = 0.088; Pap31 (IgG) = 0.212; SCS-β (IgM) = 0.056; SCS-β (IgG) = 0.107; GroEL (IgM) = 0.093; GroEL (IgG) = 0.400

**Fig 5:**
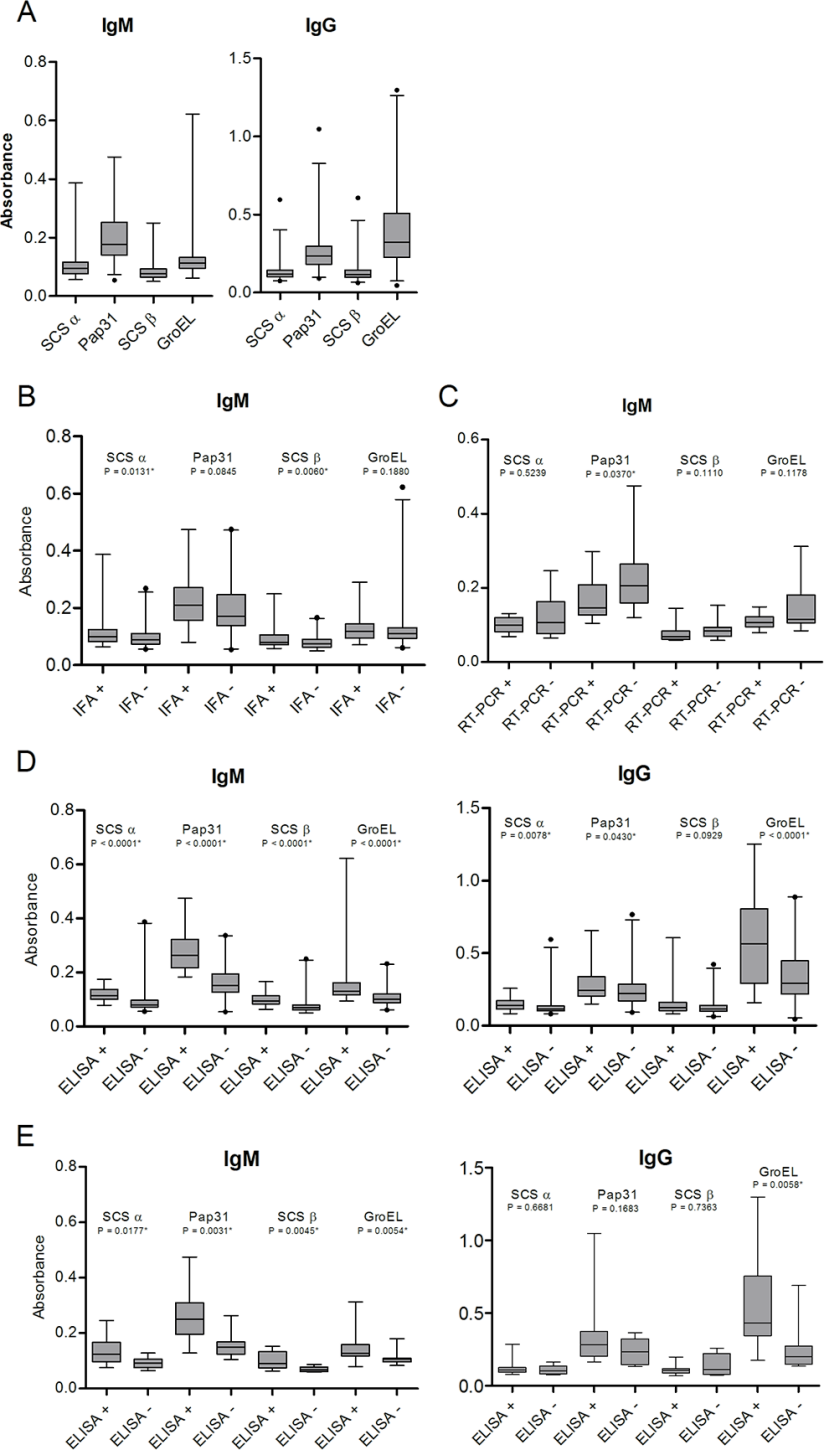
IgM and IgG levels for antigenic candidate ELISAs. A) Overall IgM and IgG data for the study population. Data are presented as boxplots that illustrate the medians and the 1 and 99% percentiles. Outliers are marked with circles. B) IgM ELISA results of post-outbreak localities for SCS-α, Pap31, SCS-β and GroEL according to IFA results. C) IgM ELISA results of post-outbreak localities for SCS-α, Pap31, SCS-β and GroEL according to RT-PCR results. D) IgM and IgG ELISA result of post-outbreak population for SCS-α, Pap31, SCS-β and GroEL taking into account the whole cell *B*. *bacilliformis* ELISA assay. E) IgM and IgG ELISA result for the endemic area (Huancabamba) for SCS-α, Pap31, SCS-β and GroEL according to the whole cell *B*. *bacilliformis* ELISA assay. For B, C, D and E data are presented as boxplots that illustrate the medians and the 25th and 75th quartiles, and the whiskers represent the 1% and 99% percentiles. Outliers are marked with circles. *P*-values were calculated using the Mann-Whitney test.

Fig 5 shows the IgM and IgG levels for antigenic candidate ELISAS for all the study population (Fig 5A). Moreover, Fig 5B and 5C show the IgM results of post-outbreak localities for each antigen ELISA according to the IFA and RT-PCR results (statistically significant differences for SCS-α and SCS-β according IFA results and Pap31 according RT-PCR results). No significant differences were found for IgG when the IFA or RT-PCR results were taken into account. Fig 5D and 5E represent the results of the antigenic candidate ELISAs according to the whole cell *B*. *bacilliformis* ELISA assays for the post-outbreak areas (Fig 5D) and the endemic area (Huancabamba) (Fig 5E). It is of note that in the case of IgM, concordance between the ELISAs for antigenic candidates and the ELISA for whole cell *B*. *bacilliformis* was very high and ranged from 98.3 (59/60) to 100% (60/60). By comparing the antigenic candidates ELISA results between the post-outbreak villages with the endemic area, only the IgG levels of SCS-α were significantly higher in the post-outbreak areas than in Huancabamba (*P* = 0.0225).

It seems that the positive samples for IgM ELISA of any of the antigenic candidates tested show a positive correlation with the remaining candidates (*P* varies from <0.0001 for SCS-α / GroEL to 0.0041 for SCS-β / GroEL). Regarding IgG ELISA of the antigenic candidates, only GroEL did not show a significant association with the remaining antigenic candidates. Nonetheless, a possible bias related to the antigenic-candidates selection process cannot be ruled out, and thus a posterior analysis using samples from different endemic areas are needed to perform an in depth analysis.

## Discussion

Currently, early diagnosis of Carrion’s disease remains an unsolved problem due to the socio-geographical context of the endemic areas; the illness is present in poor, rural and isolated areas with precarious access, with neither the necessary equipment nor qualified personnel to perform molecular/immunological techniques. In endemic areas, the diagnosis is usually based on clinical symptoms or thin blood smear, an expertise-dependent methodology with 96% of specificity but very low sensitivity (24–36%) [19]. This problem is further enhanced by the unspecific initial symptoms of Carrion’s disease, common to a several pathologies existing in these areas, such as dengue and other arboviral diseases, malaria or tuberculosis [7,20]. All of this leads to misdiagnosis of patients [20,21] and the non treatment of asymptomatic carriers, thereby perpetuating disease transmission. The present data highlight the non-concordance between symptomatology and antibody levels. This is a relevant fact emphasizing again the challenges that exist in diagnosing the illness clinically. Bacterial culture is not clinically useful due to the culture requirements and the slow bacterial growth rate [6,20]. Molecular and serologic tools, like PCR or IFA are able to detect acute cases more efficiently but are very difficult to be implemented in routine practice in remote endemic rural areas [1,8,22]. The use of sensitive and specific rapid diagnostic tools is a way to overcome these limitations.

Chamberlin *et al*. described that 82% of acute confirmed cases and 91% of convalescents showed a positive IFA test, while 74% of the volunteers with a positive IFA test had had Bartonellosis within the last year. This percentage decreased to 40% when a more distant or a non-bartonellosis episode was reported [8]. Accordingly, the IFA positivity from the samples collected in the established endemic area in our study were the lowest at 12.5%. Nonetheless, only 26.8% of the individuals from post-outbreak zones had a positive result in the IFA test. This, together with Chamberlin’s results, suggest that IFA positivity peaks just after clinical resolution and slowly decreases thereafter. However, other factors could affect the final IFA positivity and should be taken into account, including the *B*. *bacilliformis* strain used for its development, and its specific levels of antigen expression as well as the subjectivity associated with the reading of the slides. In fact, in a previous study developed by Chamberlin *et al*, differences were observed in IFA positivity when two different *B*. *bacilliformis* strains were used [8].

Previous studies have explored the use of PCR techniques to diagnose the acute phase of Carrion’s disease. Thus, del Valle *et al* [22] detected 21 acute Carrion’s disease cases analyzing 113 previously diagnosed as negative patients using a *Bartonella*-specific *16S rRNA* based PCR. Nonetheless, further studies showed the potential limitation of this technique to detect asymptomatic carriers [23]. The use of RT-PCR has also been studied [16], 14 positive acute being detected cases among 63 previously negative children. It has been shown that RT-PCR has a higher sensitivity than classical PCR procedures [24], but to the best of our knowledge the use of RT-PCR to detect asymptomatic *B*. *bacilliformis* carriers has not been performed. Nonetheless, the positive results observed were close to the detection limit of the technique, showing that the bacterial burden present in the blood of asymptomatic carriers may be extremely low, suggesting the possible presence of bacteria in specific tissues which remain apparently undetected in blood over long periods of time. Although all volunteers received a 14-day course of ciprofloxacin during the outbreak, it is noteworthy that there was still a very high number of confirmed asymptomatic carriers by RT-PCR. This suggests that the short time elapsed since the outbreak might have favored the high number of positive samples by RT-PCR. Despite a case in which the duration of asymptomatic bacteremia was up to 3 years [25], there is a lack of in depth studies about bacteremia in asymptomatic carriers. In any case all these results highlight the enormous relevance of the need for techniques which may be implemented in endemic areas to detect asymptomatic carriers in order to adequately control and possibly eradicate this illness.

ELISA data showed high levels of antibodies against *B*. *bacilliformis*, which was expected because an outbreak had occurred several months earlier in 4 out of 5 of the villages analyzed. It is known that inhabitants of endemic areas develop partial immunity due to exposure to *B*. *bacilliformis* [5]. Accordingly, the inhabitants from the long established endemic infected area showed significantly higher IgG levels when compared with the other 4 non-endemic villages together or with each of the post-outbreak villages. Similarly, IgM and IgG levels were age-dependent suggesting the development of partial immunity throughout life. The IgM levels were significantly higher when comparing individuals ≤25 years-old with adults ≥26 years. Moreover, the IgG levels were significantly lower for the age group ≤10 years-old when compared with each of the age groups of adult population (≥11 - ≤25; ≥26 - ≤55 and ≥56 - <70) with the exception of individuals ≥70 years. Regarding the differences in the antibody levels by gender, the IgG levels were higher in men probably because men often move to the neighboring endemic areas such as Cajamarca department (San Ignacio area) to work in the coffee bean harvesting. The relation between coffee plantations and the presence of *Lutzomyia* spp. has already been reported [26].

The presence of antibodies and the blood carriage of *B*. *bacilliformis* open the door to different possibilities such as the bacterial evasion of the host immune system by changes in the expression of bacterial epitopes which may correlate with decreased ability to invade erythrocytes and facilitate bacterial trapping in other tissues.

In the present study we have identified four potential antigenic candidates, two of which have previously been reported in the literature, GroEL and Pap31 [9,10,13], while the remaining two are described for the first time. These new antigenic candidates, SCS-α and SCS-β are involved in the tricarboxylic acid cycle, an important cytosolic metabolic pathway. Indeed, subunit α has been described as an immunogenic protein of *Brucella melitensis* [27], and the β subunit was recently reported in *Bartonella henselae* pathogenesis [28].

Pap31 or Hemin-binding protein A has shown to be a good candidate in the ELISA technique and is highly expressed in *B*. *bacillifomis* cultures [13]. Furthermore, Pap31 is an outer membrane protein that seems to be good candidate for the development of serodiagnostic tools for *Bartonella* infections, as proposed for *Bartonella quintana* infections [17].

Knobloch *et al*. [10] described that GroEL, a heat shock protein, never bound to *Bartonella* IgM antibodies, suggesting that GroEL may be a good antigenic candidate for the chronic phase. Similarly, in the present study GroEL was identified as reactive with IgG. However, we were also able to detect IgM anti-GroEL. Differences in the time elapsed from the infection and the sample collection may explain this finding. GroEL is present in the outer and inner membranes of *B*. *bacilliformis*, having also been detected in *B*. *bacilliformis* supernatants. Its presence is correlated with mitogenic activity against human vascular endothelial cells which leads to the development of verrucous lesions [29]. This mitogenic activity is inhibited *in vitro* by the presence of specific anti-GroEL antibodies, suggesting the protective role of specific IgG in asymptomatic carriers. Indeed, the presence of specific IgG anti-GroEL was observed in the volunteer presenting a Verruga Peruana. GroEL has also been described as a good candidate for vaccine production and the development of diagnostic kits for *Brucella melitensis* [30].

The strong correlation between the positive results for IgM ELISAs anti-*B*. *bacilliformis* whole cell and positive results for antigenic candidates, suggests that the antigens can be used indistinctly in a rapid diagnostic test. These are preliminary results and more studies should be done to characterize these *B*. *bacilliformis* antigens.

To the best of our knowledge, to date only 5 antigenic candidates of *B*. *bacilliformis* have been described in the literature. In this study we have identified 2 new antigens of *B*. *bacilliformis*. These kind of antigens are of special relevance in the development of new, easy and cheap rapid diagnostic tools, able to be implemented in remote rural areas without the need for specific expertise. The diagnosis and treatment of both patients and asymptomatic carriers, who continue to perpetuate the illness, is crucial to reduce the burden of this disease. Therefore, studies characterizing antigens expressed during *B*. *bacilliformis* infection are fundamental to elucidate the pathogenesis of this disease and may be useful for the development of a rapid diagnostic tool, absolutely necessary to advance towards Carrion’s disease eradication.

## Supporting Information

S1 Fig The figure shows the geographical localization (district, province and department) of the study area. Three of the villages (Tunal, Guayaquiles and Mayland) are located within the Lalaquiz district, while Los Ranchos is in Canchaque district, and Huancabamba is in the homonymous district.
(TIF)
