# Supplementary figures and images for: Succinyl-CoA Synthetase: New Antigen Candidate of Bartonella bacilliformis
Source: PLoS Negl Trop Dis. 2016 Sep 14;10(9):e0004989. doi: 10.1371/journal.pntd.0004989 (PMC5023120; doi:10.1371/journal.pntd.0004989)

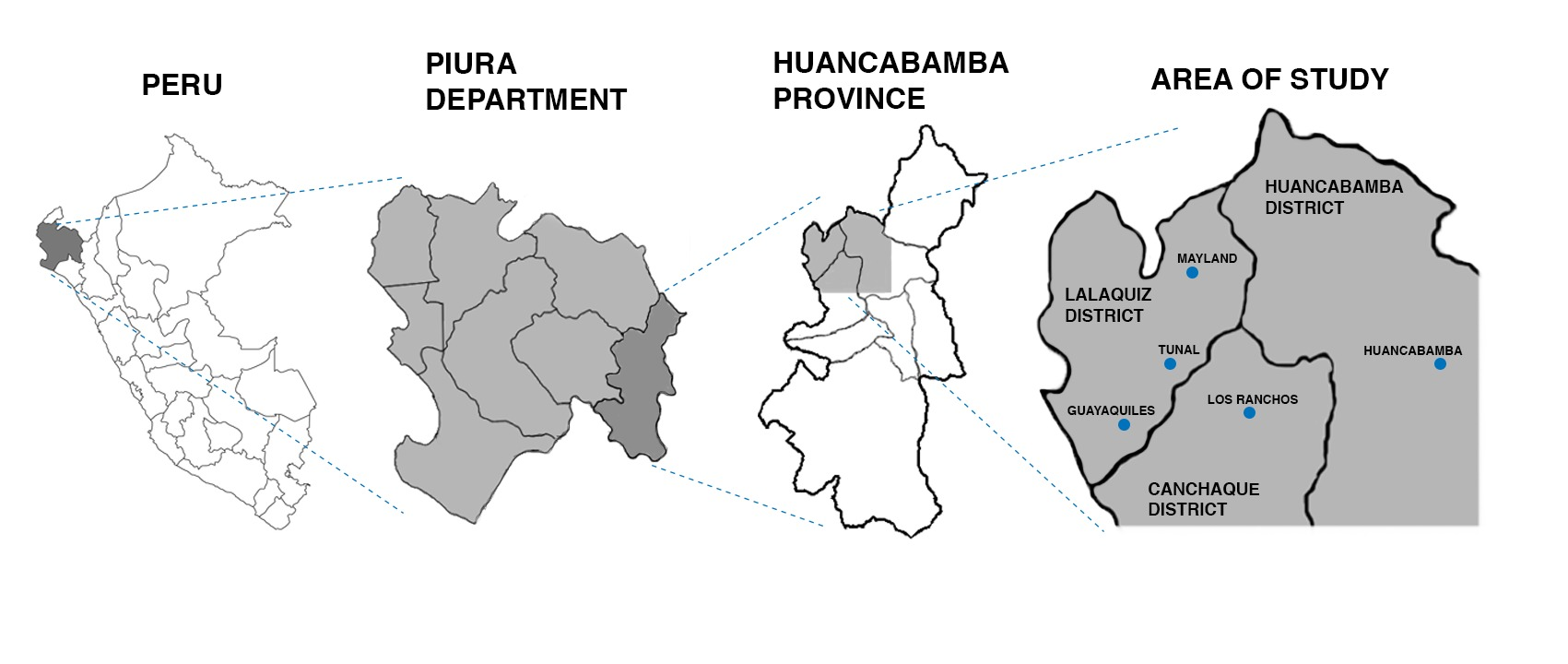

Supplement: S1 Fig — Three of the villages (Tunal, Guayaquiles and Mayland) are located within the Lalaquiz district, while Los Ranchos is in Canchaque district, and Huancabamba is in the homonymous district. (TIF) [file pntd.0004989.s001.tif]
